# A randomised controlled trial of cognitive aids for emergency airway equipment preparation in a Paediatric Emergency Department

**DOI:** 10.1186/s13049-016-0201-z

**Published:** 2016-01-27

**Authors:** Elliot Long, Patrick Fitzpatrick, Domenic R. Cincotta, Joanne Grindlay, Michael Joseph Barrett

**Affiliations:** Department of Emergency Medicine, The Royal Children’s Hospital, 50 Flemington Road, Parkville, VIC 3052 Australia; Murdoch Children’s Research Institute, 50 Flemington Road, Parkville, Australia; Department of Paediatrics, Faculty of Medicine, Dentistry, and Health Sciences, University of Melbourne, Parkville, VIC Australia; Paediatric Emergency Research Unit, National Children’s Research Centre, Crumlin, Dublin, Ireland

**Keywords:** Airway management, Intubation, Decision support systems, Quality assurance

## Abstract

**Background:**

Safety of emergency intubation may be improved by standardising equipment preparation; the efficacy of cognitive aids is unknown.

**Methods:**

This randomised controlled trial compared no cognitive aid (control) with the use of a checklist or picture template for emergency airway equipment preparation in the Emergency Department of The Royal Children’s Hospital, Melbourne.

**Results:**

Sixty-three participants were recruited, 21 randomised to each group. Equal numbers of nursing, junior medical, and senior medical staff were included in each group. Compared to controls, the checklist or template group had significantly lower equipment omission rates (median 30 % IQR 20–40 % control, median 10 % IQR 5–10 % checklist, median 10 % IQR 5–20 % template; *p* < 0.05). The combined omission rate and sizing error rate was lower using a checklist or template (median 35 % IQR 30–45 % control, median 15 % IQR 10–20 % checklist, median 15 % IQR 10–30 % template; *p* < 0.05). The template group had less variation in equipment location compared to checklist or controls. There was no significant difference in preparation time in controls (mean 3 min 14 s sd 56 s) compared to checklist (mean 3 min 46 s sd 1 min 15 s) or template (mean 3 min 6 s sd 49 s; *p* = 0.06).

**Discussion:**

Template use reduces variation in airway equipment location during preparation foremergency intubation, with an equivalent reduction in equipment omission rate to the use of a checklist. The use of a template for equipment preparation and a checklist for team, patient, and monitoring preparation may provide the best combination of both cognitive aids.

**Conclusions:**

The use of a cognitive aid for emergency airway equipment preparation reduces errors of omission. Template utilisation reduces variation in equipment location.

**Trial registration:**

Australian and New Zealand Trials Registry (ACTRN12615000541505).

**Electronic supplementary material:**

The online version of this article (doi:10.1186/s13049-016-0201-z) contains supplementary material, which is available to authorized users.

## Background

In every setting reported, non-operating room (OR) intubation is a high-risk, low frequency procedure. The adverse event rate is significantly higher and the first pass success rate significantly lower than that reported in the OR [1–11]. In addition to patient-related factors, several technical and non-technical (human) factors for this have been proposed, including: the time-critical nature of non-OR intubations, the infrequency with which they are performed, the high cognitive load on team members, the “flash teams” consisting of members who may be unfamiliar with each other and the intubation environment, the lack of standardised equipment and approach, and failure to plan for unanticipated difficulties with airway management [12–14]. Strategies aimed at minimising the risk of non-OR intubations include standardised equipment preparation through the use of a cognitive aid. Both checklists and templates have been used for this purpose. Checklists have been shown to reduce the number of items omitted during a standard induction protocol in the OR [15], and to reduce the adverse event rate of Emergency Department (ED) intubations in trauma patients [16]. Templates have been described to ensure airway equipment is available and located in a standardised position during pre-hospital emergency airway equipment preparation (EAEP) [17], though their impact on patient-centred outcomes has not been studied. Use of cognitive aids during EAEP relies on two assumptions. Firstly that standardised preparation may reduce intubation related adverse events by reducing equipment errors. Secondly that standardised preparation should always include equipment for unanticipated difficulty with airway management. This involves setting up equipment for initial and subsequent intubation attempts, for failed intubation, and for a can’t intubate can’t oxygenate (CICO) scenario during preparation for every non-OR intubation. Standardisation and planning for unanticipated difficulty during EAEP is recommended by governing bodies and academic societies [18–22]. The United Kingdom’s 4^th^ National Audit Project (NAP4) of major ED airway complications recommended that intubation equipment be standardised and that all equipment likely to be required for intubation (including rescue devices) be immediately available [18].

We aimed to compare the equipment omission rate during EAEP using no cognitive aid, using an airway checklist, or using an airway template. Standard airway cart intubation equipment recommended by the Australian Resuscitation Council (ARC) was used as a gold standard (Table 1) [23].

**Table 1:** The Australian Resuscitation Council airway cart equipment list for paediatric non-operating room intubation

Oropharyngeal airway (guedel) x 3	Bougie
Tongue depressor	Laryngeal mask airway
Laryngoscope blade x 2	Magill forceps
Laryngoscope handle x 2	Nasogastric tube
Endotracheal tube x 3	Tapes/ties
Stylet (introducer) Syringe Lubricant	Can’t intubate can’t oxygenate (CICO) kit

## Methods

**Fig. 1:**
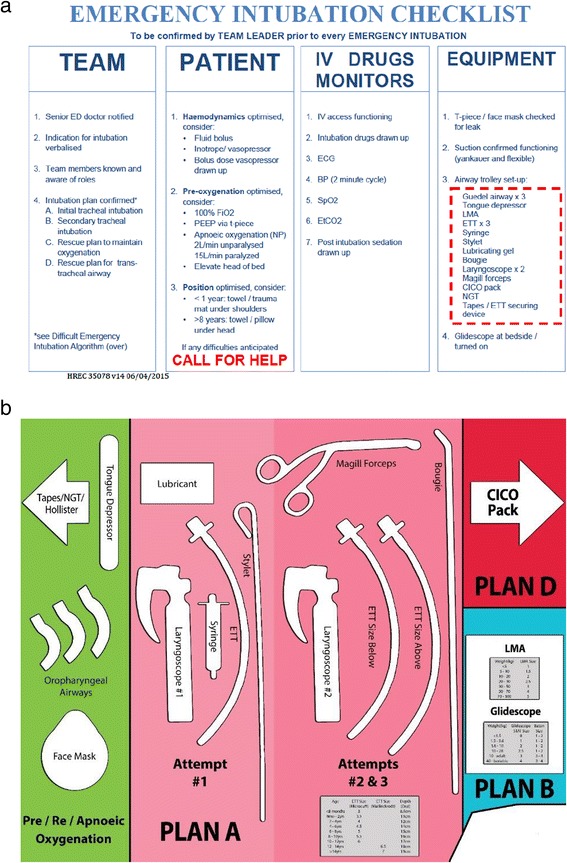
Cognitive aids used for emergency airway equipment preparation. **a** Checklist (the equipment outlined with the dotted red line on the intubation checklist was tested), **b** Template

The study was conducted in the Emergency Department (ED) of The Royal Children’s Hospital, Melbourne. The ED has an annual census of >85 000 presentations, and performs regular audit of ED intubations [5]. During routine clinical shifts, 2 ED resuscitation nurses, 1 junior and 1 senior emergency medical staff member comprise a resuscitation team caring for critically unwell or injured patients. Any of these team members are expected to be able to perform EAEP. ED resuscitation nurses receive airway education during training to work as part of the resuscitation team, and all nursing and junior medical staff participate in a half day multidisciplinary airway workshop during their ED term. Members of the resuscitation team (nursing, junior and senior medical staff) were recruited individually during clinical shifts and randomised to one of three groups: no cognitive aid (control), checklist (Fig. 1a), or template (Fig. 1b). The checklist was developed by adapting published versions [6, 24], and drafted using validated guidelines (http://www.projectcheck.org/). It included challenge-response prompts for the set-up of standardised airway equipment. The equipment template design was novel, and was undertaken by the study investigators in conjunction with the hospital’s Airway Special Interest Group. Equipment location and color-coding on the template was intended to reinforce a local Emergency Intubation Algorithm based on the Difficult Airway Societies plans A-D (http://www.das.uk.com). Equipment for pre-oxygenation and initial intubation attempts was located on the left-hand side of the template, and progressed from left to right to include equipment for subsequent intubation attempts, supra-glottic airway (SGA) rescue, and can’t intubate can’t oxygenate (CICO) kit. The template included endotracheal tube and laryngeal mask sizing charts for age/weight, and was intended to sit on top of the airway cart with equipment placed on its surface. Both the intubation checklist and template were piloted prior to inclusion in the study. This process involved feedback from all participant groups regarding layout and ease of use following simulated scenario-based patient encounters. The study was conducted in situ, in a fully equipped ED resuscitation bay. After obtaining written informed consent, participants were given identical clinical scenarios (Additional file 1) and asked to prepare the airway cart for intubation. Equipment not normally laid out on the airway cart (suction, oxygen and T-piece, medications, continuous waveform end-tidal CO2 monitor, video laryngoscope) was not included in the study. When the participant finished setting up the airway cart, their preparation time was recorded. A photograph of the airway cart was taken and the equipment prepared was manually logged on a clinical report form (Additional file 2). Equipment data was entered blindly into a study database, and equipment location was separately mapped using the un-blinded photographs. The primary outcome measure was the equipment omission rate in each study group. Secondary outcome measures included the combined errors of omission and sizing errors, the time taken for EAEP, and the location of airway equipment in each study group.

Our sample size calculation was based upon an absolute equipment omission rate of 80 % observed during an unpublished local observational study pilot. We estimated that introduction of a template or checklist would decrease in the equipment omission rate to 20 %. We aimed to recruit equal numbers nursing, junior, and senior medical staff. Using a superiority design, the study aimed to recruit 21 participants per group to deliver a power of 0.99 with an alpha of 0.05 (63 participants overall). Block randomisation using computer generated quasi-random numbers was stratified according to clinician seniority with equal distribution of strata (nursing, junior medical, and senior medical staff) per arm. The statistical analysis was based on intention to treat. Primary outcome analysis was the comparison of proportions of two independent samples using *χ*2 test. Standard deviation for normative data and interquartile ranges for skewed data were calculated.

The study was approved by the institutional ethics committee (HREC 35078a) and registered through the Australian and New Zealand Trials Registry (ACTRN12615000541505).

## Results

The equipment omission rate was significantly higher in the control group compared to the groups using a cognitive aid (checklist or template) (Table 2). There was no significant difference in omission rate between the groups using a checklist or template (*p* = 0.69). All participants in the control group omitted one or more items of equipment during EAEP. The combined rate of errors of omission and sizing errors was higher in the control group compared to the groups using a cognitive aid (checklist or template). There was no significant difference in the rate of errors of omission and sizing errors between the groups using a checklist or template (*p* = 0.32). The time taken to complete emergency airway preparation was quickest in the template group however this did not reach statistical significance. The only group to achieve 100 % completion within the time allocated in the clinical scenario (<5 min) was the template group.

**Table 2:** Errors of omission, combined errors of omission and sizing, and time taken for emergency airway preparation by study group

	Errors of omission (%); median (IQR)	p*	Errors of omission and sizing (%); median (IQR)	p*	Time to complete emergency airway preparation (min:sec); median (IQR)	p*
Control	30 (20–40)		35 (30–45)		3:09 (2:38–3:52)	
Checklist	10 (5–10)	<0.05	15 (10–20)	<0.05	3:24 (2:55–4:19)	0.6
Template	10 (5–20)	<0.05	15 (10–30)	<0.05	2:51 (2:28–3:53)	0.14

*p-values were calculated by comparing the checklist or template group with the control group

Overall, junior medical staff had the highest rate of combined errors of omission and sizing (21 % and 8 %, respectively). Nursing staff had the lowest rate of error of omission (13 %), and senior medical staff the lowest rate of sizing errors (5 %).

**Fig. 2:**
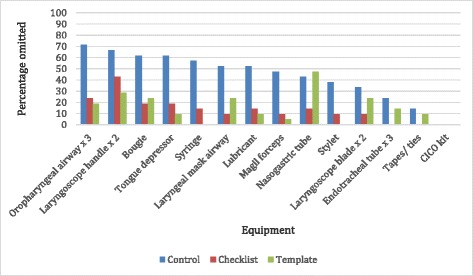
Percentage errors of omission by study group

The most commonly omitted equipment varied according to interventions used (Fig. 2). The omission rate in the control group was higher for all critical equipment, with only the nasogastric tube being omitted more frequently in the template group.

**Fig. 3:**
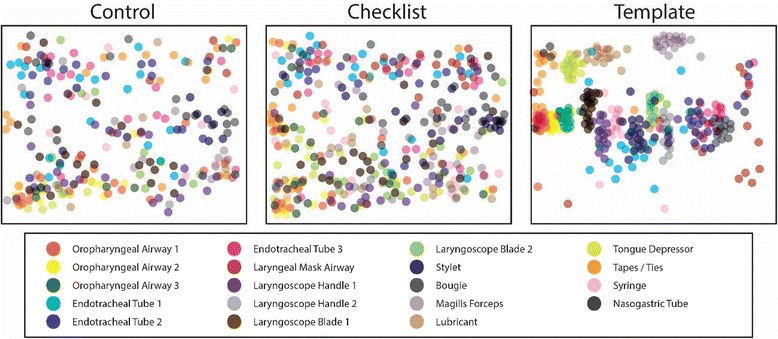
Airway equipment location by study group

Equipment location was the most consistent in the template group (Fig. 3). There was no clear pattern of equipment location in the control or checklist groups.

## Discussion

In this randomised controlled trial, we found that the use of a cognitive aid (either checklist or template) was associated with a reduction in equipment omission rates during EAEP. In addition, combined errors of omission and sizing errors were reduced, and confidence with EAEP was improved using a cognitive aid. The use of a cognitive aid was not associated with an increase in time to complete EAEP. The variation in equipment location was reduced with the use of a template.

This is the first randomised controlled trial comparing equipment omission rates during EAEP with and without the use of a cognitive aid. Prior studies have shown a reduction in the intubation equipment omission rate in the OR setting [15], and a reduction in the adverse event rate during ED intubations of trauma patients after the introduction of a pre-intubation checklist into clinical practice [16]. The most common equipment omissions in these studies included: back-up laryngoscope handles, stylets, and alternative ETT sizes. In our study, the most commonly omitted items were: alternative oropharyngeal airway sizes, a back-up laryngoscope handle, and a bougie. Before and after quality improvement studies aiming to improve the safety of non-OR intubations have demonstrated a reduction in adverse event rates through the use of protocolised checklist-driven care bundles [25]. Additionally, intubation process measures, such as non-technical (human) factors, have been improved through checklist-driven protocols for rapid-sequence intubation (RSI) in trauma patients in the ED [26] and medical Intensive Care Unit (ICU) patients [27]. These interventions address more than EAEP, though standardised equipment preparation may contribute to their effectiveness.

This study is generalisable to most non-OR intubations. Similar technical and non-technical factors exist between ED, ICU, medical emergency team, and pre-hospital environments, making standardised equipment preparation valid across these areas. For study purposes we used standard intubation equipment recommended by the ARC, though this could be modified for local use.

It was not the purpose of this study to demonstrate superiority of one cognitive aid over the other. Both the use of a checklist and template resulted in similar rates of equipment omission, though the equipment location was less variable using the template. The intubation checklist may, however, contain challenge-response prompts to address more than the equipment laid out on the airway cart, including team and patient factors, monitoring and medication factors, as well as equipment not routinely laid out on the airway cart (such as suction, oxygen, face-mask, and video laryngoscope). The airway template may be used in conjunction with the intubation checklist by incorporating “template completion” as a challenge response item. This may speed up the equipment checking process by “batching” the equipment listed on the airway cart. As demonstrated by the percentage errors of omission by study group (Fig. 2) the design of the cognitive aid clearly impacts on the type of omissions that result. In particular, the high rate of nasogastric tube omissions in the template group may have resulted from unclear equipment prompts on the template, which may be addressed by template refinement.

## Limitations

Some factors may have influenced the internal validity of this study. The intubation checklist used during the study had been in routine use for over one year at the time the study was conducted. The template, however, was introduced solely for the purposes of the study. This lack of familiarity may have resulted in poorer performance of those participants randomised to the template group. The CICO kit was routinely located on the side of the airway cart and was therefore never omitted in any study group. The study involved a simulated scenario, and therefore may not reflect how participants would perform during real scenarios. The study was, however, designed to simulate real ED pre-notification of patient arrival with a potential airway emergency. EAEP under these circumstances should occur regardless of whether the equipment ends up being used or not. We believe therefore that the study reflects real-life EAEP in the ED.

The predicted omission rate in the control group was much lower than anticipated by the local observational study pilot (80 % predicted vs 30 % observed). This may be explained by the Hawthorn Effect [28] and/or simulation artefact. Improved control group performance may have resulted in an underestimate of the effect size of the cognitive aids. Conversely, the presence of a cognitive aid does not ensure that it is used in clinical practice [29].

This study focussed on equipment preparation for non-OR intubation. Other aspects of non-OR intubation, such as patient, team, medication, and monitoring aspects of EAEP, were beyond the scope of this study. Though our results showed a clear improvement in the process of EAEP through the use of cognitive aids, it remains unclear whether this may translate into improved patient-centred outcomes.

## Conclusion

Standardised airway equipment preparation using a cognitive aid may make emergency intubation a safer procedure. Further studies might examine the impact of standardised preparation on patient-centred outcomes.

## Additional files

Additional file 1:
**Clinical scenario used by all participants for emergency airway equipment preparation.** (DOC 201 kb) Additional file 2:
**Clinical report form used for study data collection.** (DOCX 73 kb)

## Abbreviations

ARC: Australian Resuscitation Council
CICO: can’t intubate can’t oxygenate
EAEP: emergency airway equipment preparation
ED: Emergency Department
ETT: endotracheal tube
ICU: Intensive Care Unit
NAP4: 4^th^ National Airway Project
OR: operating room
