# Supplementary material for: A randomised controlled trial of cognitive aids for emergency airway equipment preparation in a Paediatric Emergency Department
Source: Scand J Trauma Resusc Emerg Med. 2016 Jan 27;24:8. doi: 10.1186/s13049-016-0201-z (PMC4730650; doi:10.1186/s13049-016-0201-z)
Supplement: Additional file 1: — Clinical scenario used by all participants for emergency airway equipment preparation. (DOC 201 kb) [file 13049_2016_201_MOESM1_ESM.doc]

**TEMP-T study pre-amble and vignette**

Thank-you for agreeing to take part in the TEMP-T study. The aim of the study is to determine whether cognitive aids help in the preparation of intubation equipment on the airway cart. We will be studying the content, layout, and timing of your airway equipment set-up. The TEMP-T study does NOT cover the preparation of all intubation equipment (suction / oxygen / end-tidal CO2 etc), it only covers the equipment that would normally be prepared on the airway cart.

All equipment needed is in its usual location. You may take out any equipment you need, and may use any aids normally available. You don’t need to prepare drugs, oxygen, suction or the Glidescope. Any equipment not physically placed on top of the trolley will not be recorded as setup.

You are working a resuscitation shift at The Royal Children’s Hospital as part of the airway team. You receive a phone call from the Metropolitan Ambulance Service. They are bringing in a 4 year old boy with status epilepticus. Seizures have continued for 30 minutes despite 1 dose of IM and one dose of IV midazolam. He has developed apnoea and is being bag-valve-mask ventilated. Sats 95%, HR 140, BP not recorded. ETA 5 mins. Please prepare the airway cart in anticipation of intubation.

You have a few minutes to familiarize yourself with equipment location and the cognitive aid (if you have been randomised to use one). Please ask any questions of the study investigator during this time.

Please let the study investigator know when you are ready to commence and once you have completed the airway trolley set-up.
