# Supplementary material for: A randomised controlled trial of cognitive aids for emergency airway equipment preparation in a Paediatric Emergency Department
Source: Scand J Trauma Resusc Emerg Med. 2016 Jan 27;24:8. doi: 10.1186/s13049-016-0201-z (PMC4730650; doi:10.1186/s13049-016-0201-z)
Supplement: Additional file 2: — Clinical report form used for study data collection. (DOCX 73 kb) [file 13049_2016_201_MOESM2_ESM.docx]

1. Study number

To be completed by study investigator

Investigator initials: ___ ___

1. Randomisation Code
2. Candidate Information ( tick one box)

Consultant/Fellow

Registrar

Nursing

1. Time to airway setup completion (xx mins yy seconds)

**Equipment checklist (circles and insert numbers)**

N

Y

Oropharyngeal airway Quantity Sizes

N

Y

Endotracheal tubes Quantity cuffed Sizes

Quantity uncuffed Sizes

N

Y

Laryngeal mask Quantity Sizes

N

Y

Larnygoscope handle Quantity

N

Y

Laryngoscope blades Quantity straight Sizes

Quantity curved Sizes

N

Y

Stylet Quantity Sizes

N

Y

Bougie Quantity Sizes

N

Y

Magills Quantity Sizes

N

Y

Lubricating gel

N

Y

Tongue depressor

N

Y

Tapes/ties/ET holster

N

Y

Syringe

N

Y

Trans trach airway kit

NGT

N

Y

Please take a photograph to identify the position of the equipment Completed (tick)

Name of data collector (BLOCK LETTERS) _____________________________
